# Concurrent infections by all four dengue virus serotypes during an outbreak of dengue in 2006 in Delhi, India

**DOI:** 10.1186/1743-422X-5-1

**Published:** 2008-01-09

**Authors:** Preeti Bharaj, Harendra S Chahar, Anubhav Pandey, Kavita Diddi, Lalit Dar, Randeep Guleria, Sushil K Kabra, Shobha Broor

**Affiliations:** 1 Department of Microbiology, All India Institute of Medical Sciences, New Delhi, India; 2 Department of Medicine, All India Institute of Medical Sciences, New Delhi, India; 3 Department of Pediatrics All India Institute of Medical Sciences, New Delhi, India

## Abstract

**Background:**

Co-circulation of multiple dengue virus serotypes has been reported from many parts of the world including India, however concurrent infection with more than one serotype of dengue viruses in the same individual is rarely documented. An outbreak of dengue hemorrhagic fever/dengue shock syndrome (DHF/DSS) occurred in and around Delhi in 2006. This is the first report from India with high percentage of concurrent infections with different dengue virus serotypes circulating during one outbreak.

**Results:**

Acute phase sera from patients were tested for the presence of dengue virus RNA by RT-PCR assay. Of the 69 samples tested for dengue virus RNA, 48 (69.5%) were found to be positive. All the four dengue virus serotypes were found to be co-circulating in this outbreak with DENV-3 being the predominant serotype. In addition in 9 of 48 (19%) dengue virus positive samples, concurrent infection with more than one dengue virus serotype were identified.

**Conclusion:**

This is the first report in which concurrent infections with different dengue virus serotypes is being reported during an outbreak from India. Delhi is now truly hyperendemic for dengue.

## Background

Dengue is one of the notable viral infections, the global epidemiology of which has changed dramatically in the past 50 years especially during the last 20 years in the tropical regions of the world. Dengue is a major public health problem worldwide, especially in the tropical and subtropical areas with around 2.5 billion people living in areas at risk [1,2]. Dengue viruses (genus *Flavivirus*, family *Flaviviridae*) are mosquito borne and the principal vector (*Aedes aegypti*) is a day-biting domestic mosquito of public importance that breeds in natural or artificial water [3,4]. Dengue illnesses are caused by any of the four serologically related viruses designated as DENV-1, DENV-2, DENV-3 and DENV-4 [5]. Infection with any one of these serotypes mostly causes a mild, self limiting febrile illness (classical dengue fever (DF)), however, a few cases develop severe life threatening dengue hemorrhagic fever (DHF) and dengue shock syndrome (DSS). The estimated number of 50–100 million infections per year results in 250,000–500,000 cases of DHF and 25,000–50,000 deaths each year [6].

The routine laboratory diagnosis of dengue virus infection is primarily achieved by the isolation of virus in tissue culture, serodiagnosis by detection of IgM/IgG antibodies and/or molecular detection by the demonstration of viral RNA by RT-PCR [7-9].

Dengue is endemic in both urban and semi urban areas of India and was first isolated in India in 1945 [10,11]. Delhi situated in the northern part of India, had outbreaks of dengue due to different dengue virus types in 1967, 1970, 1982, 1988, 1996 and 2003 [12-19]. All four dengue virus types circulate in India and cause epidemics, but only occasional cases of DHF/DSS were reported from Delhi till 1996. A large outbreak of DHF/DSS occurred in Delhi in 1996 by DENV-2 [20].

In recent years co-circulation of multiple dengue virus serotypes is being increasingly reported with concurrent infections. However the association of concurrent infections with severe forms of disease (DHF/DSS) needs further studies [21-26]. From India there is only one study in which we reported two cases of dual infection with DENV-1 and DENV-3 serotypes [27].

In this study we are reporting the results of the detection of dengue virus RNA by RT-PCR during the dengue outbreak that occurred in and around Delhi in 2006. The main feature of this report is the co-circulation of all 4 dengue virus serotypes and high percentage of concurrent infections.

## Results and discussion

### Patients, materials and methods

A total of 69 blood samples were collected from patients with <7 days history of fever presenting to outpatient departments, emergency services and indoor services of All India Institute of Medical Sciences hospital, New Delhi between mid August–November 2006. These blood samples were sent on ice to the Virology laboratory for detection of dengue viruses. The clinical basis for diagnosing the patients as having dengue virus infection was based on WHO definitions [28]. Since these were diagnostic samples received during an outbreak, no prior ethical clearance was required. However the patient information was de-linked from sample information to protect the privacy of the patients.

Viral RNA was extracted from serum samples using the automated MAGNA Pure compact nucleic acid isolation system (Roche, Switzerland) or QIAamp Viral RNA mini kit (Qiagen, Germany) as per manufacturers' instructions. Extracted RNA was stored at -70°C or used for RT-PCR immediately.

Published primers by Lanciotti et al were used [29] in this study. The viral RNA was reverse transcribed to cDNA using Avian Myeloblastosis Virus Reverse Transcriptase enzyme (AMV RT), (Promega Corp., USA) and the dengue virus downstream consensus primer (D2), common to all four dengue serotypes. Briefly, for each 25 μl reaction, 10 μL RNA and 50 pM of primer DENV-2 along with 200 μM of each deoxynucleoside triphosphate (dNTP) (Promega Corp., USA) and 20 units of AMV RT were used. The samples were incubated for 90 min at 42°C followed by heating at 70°C for 15 min to inactivate the enzyme.

For DNA amplification, 3 μl of first-strand cDNA reaction was amplified in a total volume of 25 μl containing 20 pM of each forward and reverse primer, 200 μM each of the four dNTPs (Promega, Madison, WI), 3 units of the *Taq *polymerase (Bangalore Genei, India) and 1.5 mM MgCl_2_. The reactions were allowed to proceed in an thermocycler gene Amp PCR System 9700 (ABI, USA). Dengue virus typing was done by second-round amplification with type-specific primers. Thermal cycling was performed and specific band size for each dengue virus serotypes observed as described earlier [29]. PCR products were visualized under a digital gel documentation system (Bio-Rad, UK).

### Statistical analysis

The data in this study are expressed as mean ± SD.

## Findings

**Figure 1:**
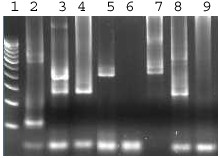
Agarose gel analysis of the product from RT-PCR followed by second-round nested PCR of RNA samples. From left to right, lane 1: 100 bp molecular marker, lane 2: sample positive for DEN-2 (119 bp), lane 3: sample with concurrent infection of DEN-3 and DEN-4, lane 4: sample positive for DEN-3 (290 bp), lane 5: sample positive for DEN-4 (392 bp), lane 6: negative sample, lane 7: sample positive for DEN-4 (392 bp), lane 8: sample positive for DEN-3 (290 bp) and lane 9: negative control.

Of 69 samples, 48 (69.5%) tested positive for dengue viral RNA by RT-PCR. Thirty-nine cases had single DENV serotype infection and 9 had concurrent infection with two DENV serotypes (see Figure 1). Of 39 single infection cases, 26 were typed as DENV-3, 9 as DENV-1, 3 as DENV-2 and 1 as DENV-4 respectively. DENV-3 dominated the outbreak constituting 54% of the positive samples, followed by DENV-1 (18.7%). The overall prevalence of concurrent infections was 19% (9 of 48 positive cases). Of 9 cases of concurrent infections, DENV-1 and DENV-3 virus serotype co-infection was found to constitute 44% (4 of 9) of the total concurrent infections. Other combinations were: co-infection with DENV-1 and DENV-4 (2 of 9, 22%), DENV-2 and DENV-3 and DENV-3 with DENV-4 (1 of 9 each, 11%). Thus, DENV-1 and DENV-3 was the most common serotype combination observed during the outbreak. Of the 48 patients in whom dengue virus RNA was detected, 28 were males and 19 females, male: female ratio being 1.52 (Table 1). One patient's laboratory proforma had no information regarding sex. Forty samples were from adults (> 12 yrs of age) and seven from children (< 12 yrs of age). The mean age of positive samples was 24.9 ± 13.8 years. The maximum number of dengue virus positive cases was in the age group of 20–30 years (35.4%) followed by the age group 12–20 years (20.8%).

**Table 1:** Age and sex wise distribution of dengue cases during 2006 outbreak.

Age	No. of Dengue cases	No. of Males	No. of Females	Ratio (M/F)
0–1	2	0	2	-
>1–5	0	0	0	0
>5–12	5	3	2	1.33
>12–20	10	6	4	1.50
>20–30	17	12	5	2.40
>30–40	8	3	5	0.60
>40	5	4	1	4
Total	47+1*	28	19	1.52

* No information regarding sex of 1 patient

The maximum number of cases reported to the laboratory for the detection of dengue virus RNA or specific IgM antibody was during the month of October 2006 in this outbreak, with a rapid decline at the onset of winter (November).

Of the 48 patients in whom dengue virus RNA was detected, the following clinical features were seen at the time of presentation: fever in all 48 (100%), low platelet count [<100,000] in 20 (41.6%), hemorrhagic manifestations and rash in 6 each (12.5%). As per classification based on WHO definition (28), DF was seen in 21 cases with single serotype infection and in 3 cases with concurrent infections, whereas DHF was present in 14 cases with single serotype infection and 6 with concurrent infections. Clinical data was inadequate in 4 cases to reach any conclusion about the severity of illness.

By far, to the best of our knowledge, this is the first report on the prevalence of concurrent infections by different dengue virus serotype combinations from an outbreak in India.

## Discussion

With the changes in the global epidemiology of dengue during the last 50 years [6], not only the number of countries reporting dengue has increased but also the number of severe disease in the form of DHF/DSS is being increasingly reported. The first epidemic of dengue in India occurred in Kolkata in 1963–64 [12] and ever since the epidemiology of dengue virus has been changing. Till 2003, Delhi was hypoendemic for dengue; however in 2003 for the first time all four dengue virus subtypes were found to co-circulate in Delhi thus changing it to a hyperendemic state (19). We have earlier reported 2 cases of concurrent infections with DENV-3 and DENV-1 in 2005 [27].

The first case of concurrent infections with 2 dengue virus serotypes was reported from Puerto Rico in 1982 [30], since then different countries have reported the occurrence of concurrent infections [21-26] in areas where multiple dengue virus serotypes co-circulate. In this study we are reporting a high percentage (19%) of concurrent infections during an outbreak of dengue in Delhi in 2006. Previous studies with lower percentage of concurrent infections have been reported from Taiwan (9.5%), Indonesia (11%) and Mexico, Puerto Rico and Indonesia together (5.5%) [21-26]. DENV-1 and DENV-3 were identified to be the most frequent dengue virus serotype combination occurring during this outbreak. This was followed by the DENV-1 and DENV-4 serotype combination. Although DENV-4 was not commonly detected as single infection, it was seen in three cases of concurrent infections. Since the predominant virus circulating during this outbreak were DENV-3 and DEN-1, the number of DENV-4 infections seen as single serotype was less as compared to DENV-3 or DEN-1.

It has been postulated that concurrent infections by multiple dengue virus serotypes may influence the clinical expression of the disease. This is considered as a single major factor for the emergence of DHF [25].

During the dengue outbreaks of 1967, 1970 and 1982 in Delhi, no culture-confirmed cases of DHF/DSS were reported [12,13,15,16]. However, some cases of DHF were seen for the first time in 1988 [17]. During the major dengue outbreak of 1996, virus isolation on 149 samples received at AIIMS hospital revealed the presence of virus in 27 samples. Of these 27 patients, DHF/DSS was seen in 59% (16/27) and DF in 40% (11/27) cases of DF. In 2006, the number of cases with DHF was 66.6% (6/9) in concurrent infections as compared to 29% (14/48) in single infection cases [19]. Thus a higher percentage of cases with concurrent infections had severe disease, though the numbers are small and larger studies are needed to prove this association.

The seasonal trend of dengue virus infection is reflected by the peak of positive cases observed during post monsoon season i.e. September–October which is in concordance with previous outbreaks [19,20]. The highest number of cases that were positive for dengue virus by RT-PCR was in the age group of 20–30 years (35.5%) as seen in Table 1. The same was also true for cases diagnosed as dengue positive by MAC-ELISA (Data not shown).

The most challenging problem associated with patient management in dengue infection is rapid diagnosis. Although the commercially available MAC ELISAs offer improvements over other conventional assays for the diagnosis, however they do not offer serotype specific diagnosis. Diagnosis based on detection of IgM antibodies can only be achieved after 5–7 days of illness. In recent years serological assay based on NS1 antigen detection have become commercially available which claim to provide early diagnosis of dengue within first day of illness. However, these tests are expensive to be used in developing countries. Moreover, a number of these newly developed kits yet need to be validated for dengue virus detection in clinical settings. The detection of concurrent infections can be made by virus isolation in tissue culture followed by indirect immunofluorscence (IFA) using serotype specific monoclonal antibodies and/or RT-PCR [31-33]. However, RT-PCR offers accuracy and speed along with serotype specific diagnosis of various circulating dengue viruses and information about co-circulation of different subtypes. In this study the samples that were received before 7 days of illness were tested by RT-PCR for detection of dengue virus RNA. An advantage of using the automated Magna Pure NA workstation was that the turnaround time for RNA extraction was considerably reduced. Further being a closed system, the probability of cross contamination between samples was greatly minimized.

It is now notably clear that the epidemics caused by multiple dengue virus serotypes, have become more frequent on a global basis in the past 18 years [6]. General belief is that concurrent infections by different dengue serotypes occur during epidemics only, where multiple virus serotypes are being transmitted. The co-circulation of multiple dengue serotypes in the same region has been documented in several countries for decades [21-26].

The detection of dengue virus RNA by RT-PCR showed that DENV-3 was the most common etiologic agent followed by DENV-1 in this outbreak. All the four dengue virus serotypes were found to co-circulate in the current outbreak of 2006; DENV-3 remained the predominant serotype as reported in 2003–2004 [20,33].

Previously from Delhi and its surrounding areas, only two concurrent dengue cases were identified [27] but now the number has significantly increased. One fact that may be of considerable importance in concurrent infections is the occurrence of recombination events [34]. Their recombination may lead to the emergence of more virulent strains.

## Conclusion

This study reveals that DENV-3 has dominated this outbreak even though all four serotypes were found to be co-circulating as detected by RT-PCR. The increasing trend of co-circulation of dengue virus serotypes suggests that Delhi is becoming a hyperendemic state from an endemic one. Although the sample size in this study was small, the study highlights a high percentage of concurrent infections by different dengue virus serotypes for the first time from India.

## Competing interests

The author(s) declare that they have no competing interests.

## Authors' contributions

PB carried out all the RT-PCR experiments, analyzed them and drafted the manuscript. HSC carried out RNA extraction, helped in careful collection of case history and drafted the manuscript. AP and KD were responsible for collection and storage of clinical samples and carried out the clinical sample processing. RG and SK clinically diagnosed the patients and directed the patients to the Virology diagnostic laboratory. SB and LD conceived the study and all the research was carried out in the Virology diagnostic laboratory under SB supervision. All authors read and approved the final manuscript.
